# Retraction Note: Strategical selection of maintenance type under different conditions

**DOI:** 10.1038/s41598-026-49957-3

**Published:** 2026-04-28

**Authors:** Mohammad M. Hamasha, Ala H. Bani-Irshid, Sahar Al Mashaqbeh, Ghada Shwaheen, Laith Al Qadri, Mohammad Shbool, Dania Muathen, Mussab Ababneh, Shahed Harfoush, Qais Albedoor, Adnan Al-Bashir

**Affiliations:** 1 Department of Industrial Engineering, Faculty of Engineering, The Hashemite University, Zarqa, 13133 Jordan; 2 Department of Industrial Engineering, Faculty of Engineering, The University of Jordan, Amman, 11942 Jordan

Retraction of: *Scientific Reports* 10.1038/s41598-023-42751-5, published online 20 September 2023

The Editors have retracted this Article.

After publication, concerns were raised that some of the references appear not to exist. Further review by the Editors additionally found that the study, which self-describes as a systematic review, lacks elements expected from a systematic review, such as well-defined search and inclusion criteria. The review is also insufficiently sourced with many of its broad statements supported only by singular references. References 58–62, which are supposed to be the source of data in Figure 3, do not exist. The Editors therefore have lost confidence in the claims of this Article.

Mohammad M. Hamasha and Mohammad Shbool disagree with this retraction. Ala H. Bani-Irshid, Sahar Al Mashaqbeh, Ghada Shwaheen, Laith Al Qadri, Dania Muathen, Mussab Ababneh, Shahed Harfoush, Qais Albedoor, and Adnan Al-Bashir did not respond to the Editors’ correspondence about this retraction.

